# A Group Emergency Decision-Making Method for Epidemic Prevention and Control Based on Probabilistic Hesitant Fuzzy Prospect Set Considering Quality of Information

**DOI:** 10.1007/s44196-022-00088-3

**Published:** 2022-05-24

**Authors:** Jian Lv, Qinghua Mao, Qingwen Li, Rongfu Yu

**Affiliations:** School of Economics and Management, Yanshan University, Qinhuangdao, 066004 China

**Keywords:** Epidemics, Group emergency decision-making under risk, Probabilistic hesitant fuzzy set, Cumulative prospect theory, Quality of decision information

## Abstract

Epidemics can bring huge impacts to economic operation and human health, a practical and effective emergency decision-making (EDM) method is of great significance to reduce all kinds of losses and slow the spread of epidemics. In the process of EDM, decision information is usually uncertain and vague, and the psychological behaviors and various perspectives of decision makers (DMs) should be considered. Hence, this paper develops a group emergency decision-making (GEDM) method under risk based on the probabilistic hesitant fuzzy set (PHFS) and cumulative prospect theory (CPT), in which probabilistic hesitant fuzzy prospect set (PHFPS) that combines PHFS and CPT is developed to portray the vagueness of decision information and psychologies of DMs. Moreover, experts’ creditability in evaluation criteria is generally different because of the differences of their own knowledge structures, practical experience, individual preference and so on. A formula is proposed to measure the quality of decision information provided by experts for revising the expert weights. In addition, the evaluation criteria supporting the GEDM of epidemics are given. Finally, the proposed method is demonstrated by an empirical case study of COVID-19, and the comparison analysis based on the rank-biased overlap model and the sensitivity analysis are conducted to the illustrate the validity of the proposed method.

## Introduction

**Fig. 1:**
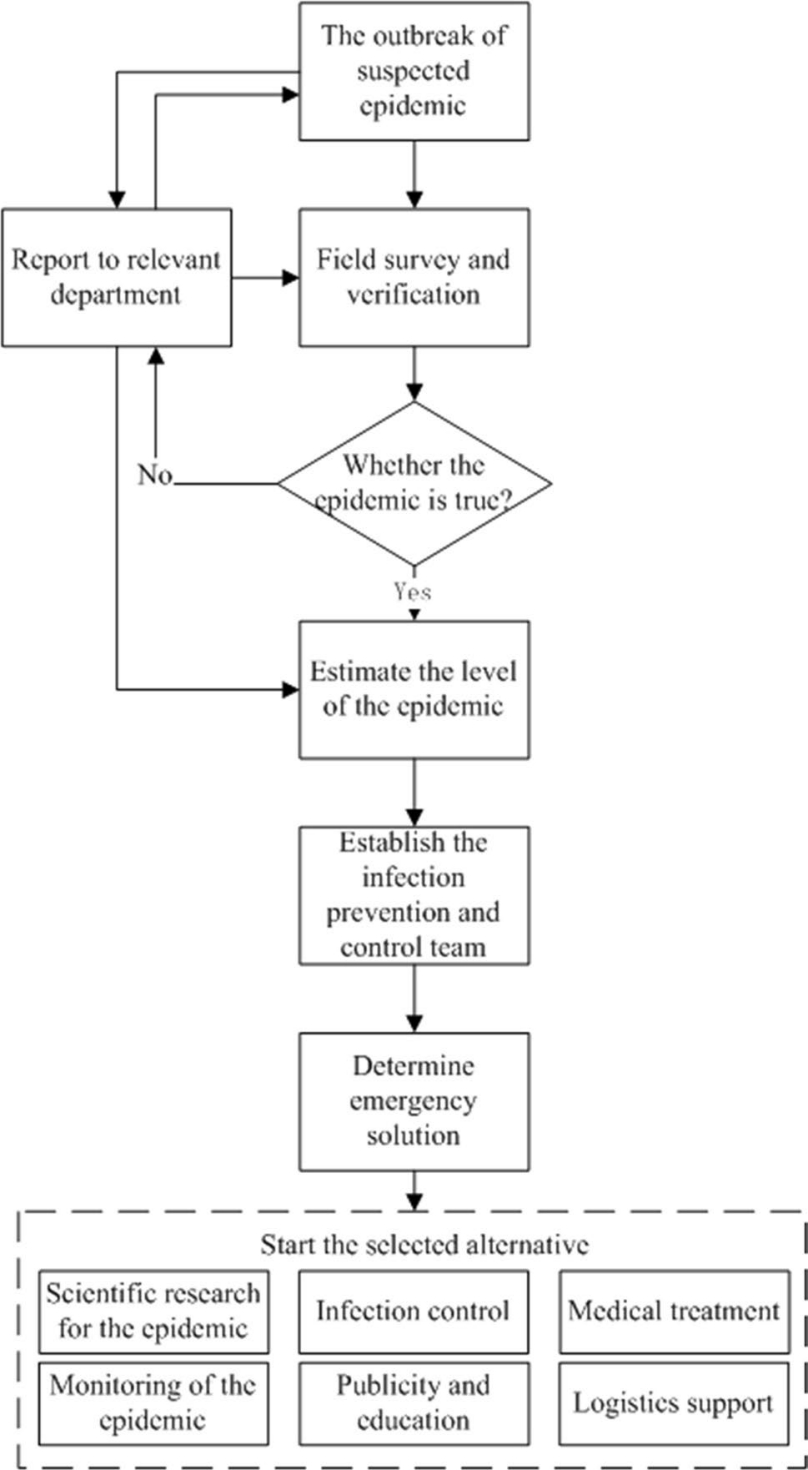
Emergency response process for an epidemic

In recent years, the frequent occurrences of the major epidemics in the world inflicted serious damages on human health, economic development and social stability, such as severe acute respiratory syndrome in 2003, Ebola virus epidemic, the coronavirus disease 2019 (COVID-19). Evidences have already indicated that effective emergency responses of government have a major part to play in flattening the epidemic curve and slowing down the arrival of peak time, which is beneficial to preventing the diagnosed patients from exceeding the capacity load of the medical system and leading to the lack of treatment resources [1]. Normally, it is difficult for the affected community or society to cope with the consequences caused by the major epidemics [2]. As a result, the research about emergency response of a major epidemic has drawn widespread attention of the governments at all levels, the scholars and the masses in today’s society. Epidemic emergency response (EER) is a complex activity, whose primary objective is to reduce or remove numerous losses and potentially harmful impacts in a sudden epidemic. A graphical illustration of the general EER process is given in Fig. 1. As can be observed from Fig. 1, determining emergency solutions, i.e., emergency decision making (EDM), is a critical component of EER. In addition, the effect of emergency solution is influenced by a great deal of factors, such as infrastructure, education level, economy and political trust. Hence, it is momentous to formulate a solution that is suitable for the epidemic disease infected area.

The sudden epidemics are characterized by rapid transmission, strong destructive power and unpredictability, which asks decision makers (DMs) to develop appropriate emergency solutions in a short time after the outbreak of an epidemic [3]. In order to improve the performance of EER, it is urgent to propose an effective epidemic EDM method. EDM methods can be divided into individual EDM or group emergency decision making (GEDM). GEDM considers various viewpoints of DMS, which contributes to make better decisions in fighting the epidemic. Usually, the GEDM process consists of two parts: (i) the information-gathering process, in which the evaluation information provided by experts is gathered and then is processed, and (ii) the selection process, where the optimal alternative is determined based on the processed information and the provided ranking model. This is in accord with the characteristics of the classic multicriteria group decision-making (MCGDM) problem.

For the past few years, various MCGDM methods have been introduced into EDM process, for instance, Ju and Wang [4] established a group decision approach combining the Dempster − Shafer theory of evidence with analytic hierarchy process and the extended Similarity to Ideal Solution (TOPSIS), in which group decision information is processed by using Dempster − Shafer theory and the extended TOPSIS for group interval data is used to obtain the ranking of the emergency alternatives. For the emergency decision-making problems with evaluation information expressed by interval numbers, Yu and Lai [5] developed a distance-based MCGDM method to deal with unconventional emergency decision-making problems. Cai et al. [6] proposed a novel emergency decision-making method that preference information of each stage is clustered by utilizing the developed similarity measurement formula for interval numbers. Liang et al. [7] presented a novel emergency decision method to select a desirable solution considering linguistic evaluations and a large number of experts from multiple groups. Zheng et al. [8] investigated the group emergency decision-making problems and constructed a dynamic method based on the case retrieval. Wang et al. [9] proposed a novel multiattribute group decision-making method with uncertain information to solve the GEDM problems, in which the hesitation of DMs and the dynamic evolution of emergency are considered.

During the actual process of epidemic emergency response, the decision information collected by DMs is usually incomplete and inaccurate due to the timeliness and ambiguity of emergency decision making. Moreover, it is universally believed that the uncertainties and the complexities of things in the real world are inevitable, and the estimation errors and inaccuracies exist in the process of measurement due to the fuzziness thought of humans and much information that is difficult to describe precisely [2, 10]. DMs are required to select the optimal solution based on the multiple, usually conflicting, criteria during the GEDM process of an epidemic. Therefore, the practical GEDM problem under epidemic situation belongs to the fuzzy MCGDM problems. Obviously, it is essential to develop some effective tools to portray the fuzziness of evaluation information in the decision-making process. With regard to this issue, fuzzy set (FS) introduced by Zadeh [11] provides a solution. To satisfy the needs of diversified application situations, many extended forms of FS were developed, such as the hesitant fuzzy set [12], hesitant fuzzy linguistic term set [13–15], the probabilistic hesitant fuzzy set [16] and so on. Linguistic variable is also an important technique to model the uncertain assessments [17]. For other forms of language variables, Zhang et al. [18] used comparative linguistic expression preference relations to express uncertain opinions of DMs in group decision making. Wu et al. [19] presented distributed linguistic representations to model the uncertainty and complexity of preference information. Zhang et al. [20] introduced personalized individual semantics into failure modes and effects analysis. It is noteworthy that all of these extended forms have unique advantages. In the complex GEDM environment, the experts hardly give accurate evaluation information. Although language variables can well express the DMs’ uncertain evaluation, it is difficult to depict the performance of all criteria. Moreover, experts may hesitate among several possible values. Probabilistic hesitant fuzzy set (PHFS) contains all possible membership degrees and the corresponding probabilities in a set to represent the performance of an object, which means that the irresolution of human and probabilistic preference will be taken into account. The theory of PHFS and its application have been developed rapidly as it can depict the vagueness more comprehensively and can express the DMs’ preference better. Therefore, PHFS is an ideal tool to describe the fuzziness of the alternative performance and the DMs’ evaluation behaviors under the real GEDM of epidemic.

At present, some scholars have introduced PHFS into GEDM. Liu et al. [21] used cumulative residual entropy to measure the level of uncertainty of probabilistic hesitant fuzzy elements in the emergency group decision-making process. Ding et al. [22] developed an extended TODIM method to solve dynamic GEDM problems, in which the fuzziness of evaluation information is portrayed by PHFS. Gao et al. [23] proposed a GEDM model that used the hesitant probabilistic fuzzy set to describe the inadequate information and the uncertainty of the external environment. The abovementioned studies afford lessons for the application of PHFS in GEDM. The experts are known to evaluate the alternatives according to multiple criteria depicted by utilizing knowledge of different fields. Due to the individual preference and limitation of knowledge, the criterion value given by an expert is more credible when the knowledge used to assess the criterion is familiar to this expert. Hence, if there is an outliers during the evaluation values with regard to the same criterion, which means that the expert giving the outliers won’t probably master this criterion well, the expert concerning this criterion is untrustworthy, and the weight of the expert should be low. For example, for a criterion of an alternative, four experts provide the evaluation values in the form of PHFS as {0.6(0.3), 0.8(0.7)}, {0.2(0.5), 0.3(0.5)}, {0.2(0.0.7), 0.4(0.3)} and {0.2(0.4), 0.3(0.6)}. It is clear that {0.6(0.3), 0.8(0.7)} deviates greatly from the average evaluation value, the influence of this evaluation value on decision making should be weakened. The most of decision-making approaches in probabilistic hesitant fuzzy environment process the decision information based on its values, which shows that these methods simply focus on the quantity of the decision information. However, information, as a very crucial element in GEDM, should be utilized fully, more specifically, it is not only necessary to concern the quantity of the decision information, but also significant to mine the data structure, which is called as the quality of the decision information. To this end, this paper constructs an effective method that takes the full data characteristics of decision information into consideration.

During the GEDM process under the epidemic situation, the DMs face the risk. Due to the uncertain evolution of epidemic, multiple possible emergency states will occur, it is difficult for DMs to determine the evolution of potential scenarios. This is consistent with fuzzy MCGDM problems under risk, the criteria values of alternatives are random variables and can vary according to emergency states. Furthermore, the future condition of the epidemic usually cannot be forecasted, but the probability distribution of the possible conditions can be obtained to quantify the randomness based on the existing data. However, the problem of MCGDM under risk with probabilistic hesitant fuzzy information has not been investigated in the existing research. Consequently, we intend to fill this gap by studying the problem of GEDM under risk in probabilistic hesitant fuzzy environment.

The methods mentioned above are mainly developed based on the expected utility theory (EUT), in which the DMs are supposed to be completely rational. The traditional MCGDM methods calculate the criteria values based on the intuitive judgments and physical data of DMs and determine the optimal alternative with the best utility score, while the study on behavior features prove that the DMs should be bounded rational rather than completely rational in the decision-making process [24, 25]. It is extremely difficult to eliminate the impact of psychological characteristics of DMs in the process of GEDM under a major epidemic. At present, several behavioral theories have been incorporated into MCGDM, such as prospect theory [26], cumulative prospect theory (CPT) [27–29], the interactive and multiple attribute decision making (TODIM) [24, 30–32], and regret theory [33, 34].

In the emergency alternative selection process, the perceived values of alternatives are determined relative to a certain reference point (RP). RP plays the part of a boundary that distinguishes gains from losses [27]. The DMs regard the excessive part as “gain” if the criteria values of the alternatives are better than the corresponding RP. In contrast, the insufficient part is deemed as “loss”. The sensitivity level of DMs on “gain” or “loss” diminishes with the distance from the RP, and DMs are more sensitive to losses than to gains because an individual is inclined to underestimate the probability of gains while overestimate the probability of losses [35]. The research shows that DMs are risk averse when facing “gain” and risk seeking while decision outcomes are classified as “loss” [36, 37]. Thus, it is important to consider psychological features of DMs when studying the emergency alternative selection behavior to avoid potential bias. CPT was introduced in 1992 by Kahneman and Tversky based on the prospect theory. It describes the way that DMs make a choice among probabilistic alternatives under risk when the probabilities of outcomes are known, which is in accord with the features of MCGDM problems under risk. The value function in CPT is used to depict the behaviors of diminishing sensitivity and loss aversion of DMs under uncertainty, and the weighting function proposed in CPT is utilized to show the characteristic reflection pattern of attitudes to risk [27]. At present, CPT has been widely applied to solve decision-making problems, such as traffic management [38, 39], portfolio insurance [40], health domains [41], EDM [35, 42, 43], and so on. Moreover, compared with other behavior theories, CPT has significant advantages in dealing with the emergency alternative selection problem under a major epidemic because its evaluation value is easy to calculate and it could describe the behavioral features of DMs in detail. Accordingly, CPT is suitable for studying the GEDM behavior by bounded rational DMs under a major epidemic with probabilistic hesitant fuzzy information.

To sum up, there are still several problems in the existing GEDM research, which makes current decisions hardly conform to the real situations of epidemic prevention and control.

1. Firstly, researchers pay less attention to the complete framework of GEDM in the context of epidemic prevention and control. An appropriate GEDM method of epidemics is essential to reduce various losses.
2. Secondly, PHFS is a powerful tool to describe the vagueness that generally exists in GEDM problems. CPT is the appropriate technique to portray the influence of DMs’ psychological behaviors on GEDM. Therefore, the combination of PHFS and CPT is constructive and meaningful. However, membership degree belongs to [0,1], it is inappropriate to take membership degree as the input of the value function of CPT. Thus, determining how to combine PHFS and CPT effectively and sufficiently is a problem facing GEDM for epidemic prevention and control.
3. Thirdly, the authority of experts on different criteria is usually different, which means that the quality of evaluation information may be unequal. How to model the authority of each expert concerning different criteria should be considered in GEDM.

Therefore, we will develop a novel GEDM method based on PHFS and CPT considering the quality of decision information, which provides insightful information for the DMs to analyze and select the optimal emergency plan of an epidemic. In this method, PHFS is used to describe decision formation, a formula is developed to determine the information quality and used to revise the expert weights. Moreover, DMs’ psychological behaviors such as loss aversion and reference dependence are investigated and a CPT-based quantitative model is provided to depict such psychologies. Furthermore, evaluation criteria for GEDM of an epidemic are given. Finally, the final evaluation values of alternatives are computed. The novel contribution of our work can be summarized as follows.

- PHFPSs, combining PHFSs and the value function of CPT, are proposed to portray the fuzziness of decision information and the psychological behaviors of DMs in the GEDM process of an epidemic. Moreover, our method takes into account possible states of an epidemic, and the weighted function is used to depict the perceived probabilities of the possible epidemic’s states. This is of great significance to solve the GEDM problem and makes the outcome more convincing and reasonable.
- This paper takes the quality of decision information into consideration. The developed models for measuring the quality of PHFS and revising the expert weights can reduce the impact of outliers given by experts and then better decision results can be obtained.
- This paper summarizes evaluation criteria for selecting an optimal emergency solution of an epidemic. A valuable reference can be gained for DMs, which may shorten the time of GEDM under a sudden epidemic. This is of practical importance.
- A whole procedure is provided to deal with GEDM problem under an epidemic, and is used to select a desirable emergency solution in fighting COVID-19 so as to illustrate the its practicability. This study introduces rank-biased overlap model to analyze the ranking results provided by different methods. This inspires the researchers who need to conduct comparison analysis with a lot of alternatives.

The rest of this article is arranged as follows. In Sect. 2, evaluation criteria system is established. Section 3 reviews the knowledge with regard to CPT and PHFS. Section 4 provides a model to measure decision information quality. Section 5 develops PHFPSs to depict the fuzziness of decision information and the psychological behaviors of DMs. In Sect. 6, the evaluation criteria are given, and the PHFPS-based GEDM method is established by revising the expert weights based on the quality of the decision information. Section 7 applies the proposed method to a real example, the comparative analysis and sensitivity analysis are conducted, and the managerial implications are given. Some conclusions and directions for future research are offered in Sect. 8.

## Establishment of Evaluation Criteria for Emergency Alternatives

In the early stage of an epidemic, governments need to quickly develop an emergency solution to avoid serious consequences on public health and the economy, and the rapid transmission of the virus implies that the governments have to carry out such a solution without much time to confer with the general public [44]. Different governments in general attach different importance to the economy, a strict and extensive epidemic emergency solution will make GDP suffer for a long period of time, hence, some governments are more willing to impose a lax emergency solution to minimize the impacts of the epidemic on their economy, while others are not, because that would lead to a rapid growth of potentially infectious cases [45]. Additionally, the research shows that significant differences across countries or areas in some characteristics, such as governmental authority, economic system, culture, educational standards and so on, which have an indelible influence on the effectiveness of emergency solution [44, 46, 47]. For instance, the peaceful protests have taken place in some countries, such as Serbia, Denmark, and Germany. In contrast, the general public in China and South Korea can better comply with the lockdown measures. Georgieva et al. [48] evaluated the containment measures by using three criteria (i.e., effectiveness, restrictiveness, and compliance). The effective implementation of the epidemic emergency plan is guaranteed based on the support of a large number of emergency medical supplies (e.g., masks, testing kits and reagents, gloves, alcohol solutions, hazard material suits, etc.), while the responses of most governments are weak in ensuring availability of much needed logistics, even if all sectors of society actively participate in the fund raising to buy supplies, the continued shortages of facilities, tools, and infrastructure for dealing with public health emergencies are difficult to be alleviated in the near future, which is detrimental to preventing the spread of an epidemic [49]. Yoo et al. [47] found that a good economic support for testing and treatment is conducive to improving the compliance of citizens to epidemic emergency plans. Altiparmakis et al. [44] provided that trust in partisanship and political leadership played a central role in assessing the measures of battling COVID-19, and there is an unquestionable link between trust and positive evaluations. Chen et al. [45] studied five potential factors that influence the satisfaction of governments’ responses to COVID-19 crisis: number of deaths per million population, number of confirmed cases per million population, stringency policies, governments’ containment and health policies, and economic support policies.

Some researchers have studied the decision-making problems under the pandemics, and we can also consult the work when establishing the evaluation criteria of epidemic emergency alternatives. For instance, Ashraf and Abdullah [50] provided eight basic public health emergency factors (e.g., increased personal protective equipment, banned intra‐city transportation and first‐aid training) to reduce the general risk of COVID-19 in the selection of preventive and mitigation measures. Jia et al. [51] analyzed the epidemic prevention and control strategies of the public, and deemed that cultural differences and irrational emotions brought a high degree of uncertainty to the prevention and control of the epidemic. Cui et al. [52] proposed a group decision-making method for the selection of a nucleic acid testing scheme, in which supplies is considered as the most important factor. Almagrabi et al. [53] established a decision-making framework to select appropriate prevention alternatives from five aspects.

Based on the existing literature for evaluation of epidemic emergency measures, several evaluation criteria for evaluating epidemic emergency alternatives are summarized in Table 1. It is clear that the criteria in Table 1 are hard to be accurately portrayed, and experts are difficult to provide completely rational evaluation values in a short time under a sudden epidemic. It is essential to use an efficient tool for describing the vagueness and the experts’ psychologies in the evaluation process.

**Table 1:** Optional evaluation criteria for emergency alternatives

No.	Criteria	Definition	References
1	Effectiveness	The ability of decreasing COVID-19 to be transmitted from person to person	Chen et al. [45], Yoo et al. [47], Georgieva et al. [48]
2	Restrictiveness	The level of restrictions on physical and social opportunities for public	Chen et al. [45], Yoo et al. [47], Georgieva et al. [48], Ayuningtyas et al. [49], Ashraf and Abdullah [50]
3	Compliance	The level of compliance with emergency alternative of public	Altiparmakis et al. [44], Armitage et al. [46], Georgieva et al. [48]
4	Emergency supplies	The capacity of supplying emergency supplies needed in the process of implementing the alternative	Yoo et al. [47], Ayuningtyas et al. [49], Ashraf and Abdullah [50], Almagrabi et al. [53], Jia et al. [51], Cui et al. [52]
5	Cost support	The level of financial support for testing and treatment	Chen et al. [45], Yoo et al. [47]
6	Economic relief	The level of debt relief (a government freezing financial obligations for households) and income support (a government providing direct cash payments to people who lose their jobs or cannot work)	Altiparmakis et al. [44], Chen et al. [45], Georgieva et al. [48]

## Preliminaries

### Probabilistic Hesitant Fuzzy Set

#### Definition 1

[16]. Let $X$ be a fixed set, a PHFS on $X$ is in terms of a function that when applied to X returns to a subset of [0, 1]. PHFS is described as $H = \left\{ {\left. {\left\langle {x,h_{x} (p_{x} )} \right\rangle } \right|x \in X} \right\}$, where $h_{x} (p_{x} )$ represents the probabilistic hesitant fuzzy element (PHFE). $h_{x}$ is the possible membership degrees of the element $x \in X$ to the set $H$, and $p_{x}$ denotes the probability of membership degree $h_{x}$, $\sum {p_{x} } = 1$.

In order to make the application of PHFS more convenient, Zhang et al. [54] expressed it as follows.

1 $$ h(p) = \left\{ {\left. {\gamma_{l} (p_{l} )} \right|l = 1,2, \cdots ,\left| {h(p)} \right|} \right\}, $$

where $\gamma_{l} (p_{l} )$ is $l{\text{th}}$ PHFE, $p_{l}$ is the probability of the membership degree $\gamma_{l}$, $\left| {h(p)} \right|$ denotes the number of PHFE in $h(p)$, and $\sum\nolimits_{l = 1}^{{\left| {h(p)} \right|}} {p_{l} } = 1$.

Some operations and aggregations can be provided according to the definition of PHFS.

#### Definition 2

[54]. Let $h_{1} (p)$, $h_{2} (p)$ and $h_{3} (p)$ be three PHFSs, then:

1. $$\varepsilon h_{1} (p) = \mathop \cup \limits_{{\gamma_{l} \in h_{1} (p)}} \left\{ {\left[ {1 - (1 - \gamma_{l} )^{\varepsilon } } \right](p_{l} )} \right\}$$;
2. $$h_{1}^{\varepsilon } (p) = \mathop \cup \limits_{{\gamma_{l} \in h_{1} (p)}} \left\{ {\gamma_{l}^{\varepsilon } (p_{l} )} \right\}$$;
3. $$h_{1} (p) \oplus h_{2} (p) = \mathop \cup \limits_{{\gamma_{{1_{l} }} \in h_{1} (p),\gamma_{{2_{a} }} \in h_{2} (p),}} \left\{ {\left[ {\gamma_{{1_{l} }} + \gamma_{{2_{a} }} - \gamma_{{1_{l} }} \gamma_{{2_{a} }} } \right](p_{{1_{l} }} p_{{2_{a} }} )} \right\}$$;
4. $$h_{1} (p) \otimes h_{2} (p) = \mathop \cup \limits_{{\gamma_{{1_{l} }} \in h_{1} (p),\gamma_{{2_{a} }} \in h_{2} (p),}} \left\{ {\left[ {\gamma_{{1_{l} }} \gamma_{{2_{a} }} } \right](p_{{1_{l} }} p_{{2_{a} }} )} \right\}$$.

#### Theorem 1

[54]. Let $h_{1} (p)$, $h_{2} (p)$, $h_{3} (p)$ and $h_{4} (p)$ be four PHFSs, let $\varepsilon$ be a positive number, and the operations in Definition 2 satisfy the following properties.

1. $$ h_{1} (p) \oplus h_{2} (p) = h_{2} (p) \oplus h_{1} (p); $$
2. $$ (h_{1} (p) \oplus h_{2} (p)) \oplus h_{3} (p) = h_{1} (p) \oplus (h_{2} (p) \oplus h_{3} (p)); $$
3. $$ \varepsilon (h_{1} (p) \oplus h_{2} (p)) = \varepsilon h_{2} (p) \oplus \varepsilon h_{1} (p); $$
4. $$ h_{1} (p) \otimes h_{2} (p) = h_{2} (p) \otimes h_{1} (p); $$
5. $$ (h_{1} (p) \otimes h_{2} (p)) \otimes h_{3} (p) = h_{1} (p) \otimes (h_{2} (p) \otimes h_{3} (p)); $$
6. $$ (h_{1} (p) \otimes h_{2} (p))^{\varepsilon } = h_{1}^{\varepsilon } (p) \otimes h_{2}^{\varepsilon } (p). $$

#### Definition 3

[54]. Let $h_{g} (p) \, (g = 1,2, \cdots ,s)$ be $s$ PHFSs, ${\varvec{w}} = (w_{1} ,w_{2} , \cdots ,w_{g} )$ is the weight vector of $h_{g} (p)$ with $w_{g} \in [0,1]$,$g = 1,2, \cdots ,s$, and $\sum\nolimits_{g = 1}^{g = s} {w_{g} } = 1$. Then, the probabilistic hesitant fuzzy weighted averaging (PHFWA) operator can be represented as follows.

2 $$ PHFWA(h_{1} (p),h_{2} (p), \cdots ,h_{s} (p)) = \mathop \oplus \limits_{g = 1}^{s} w_{g} h_{g} (p) = \mathop \cup \limits_{{\gamma_{{1_{l} }} \in h_{1} (p),\gamma_{{2_{l} }} \in h_{2} (p), \cdots ,\gamma_{{s_{l} }} \in h_{s} (p)}} \left\{ {\left[ {1 - \prod\limits_{g = 1}^{s} {(1 - \gamma_{{g_{l} }} )^{{w_{g} }} } } \right]\prod\limits_{g = 1}^{s} {p_{{g_{l} }} } } \right\}. $$

#### Definition 4

[55]. Let $h_{1} (p)$ and $h_{2} (p)$ be two PHFSs. If $\left| {h_{1} (p)} \right| \ne \left| {h_{2} (p)} \right|$, we add PHFEs to the PHFS that has fewer PHFEs. The membership degrees of the added PHFEs are the smallest in this PHFS, and the probabilities are 0. We define ${\text{MAX}}(\left| {h_{1} (p)} \right|,\left| {h_{2} (p)} \right|)$ is the bigger one between $\left| {h_{1} (p)} \right|$ and $\left| {h_{2} (p)} \right|$, then, the distance measure can be expressed by following mathematical symbols.

3 $$ d(h_{1} (p),h_{2} (p)) = \frac{1}{2}\sum\limits_{l = 1}^{{{\text{MAX}}(\left| {h_{1} (p)} \right|,\left| {h_{2} (p)} \right|)}} {\left( {\left| {\gamma_{{1_{l} }} p_{{1_{l} }} - \gamma_{{2_{l} }} p_{{2_{l} }} } \right| + \left| {\gamma_{{1_{l} }} - \gamma_{{2_{l} }} } \right|p_{{1_{l} }} p_{{2_{l} }} } \right)} . $$

#### Definition 5

[54]. For the comparation of PHFSs, we give the score function and the deviation function of a PHFS $h(p)$. The score function is defined as follows.

4 $$ S(h(p)) = \sum\limits_{l = 1}^{{\left| {h(p)} \right|}} {\gamma_{l} p_{l} } . $$

The deviation degree function is presented below.

5 $$ D(h(p)) = \sum\limits_{l = 1}^{{\left| {h(p)} \right|}} {p_{l} (\gamma_{l} - S(h(p)))^{2} } . $$

Then, an approach for comparing $h_{1} (p)$ and $h_{2} (p)$ is proposed:

1. If $$S(h_{1} (p)) > S(h_{2} (p))$$, then $$h_{1} (p) \succ h_{2} (p);$$
2. If $S(h_{1} (p)) = S(h_{2} (p))$ and $D(h_{1} (p)) > D(h_{2} (p))$, then $h_{1} (p) \prec h_{2} (p)$;
3. If $S(h_{1} (p)) = S(h_{2} (p))$ and $D(h_{1} (p)) = D(h_{2} (p))$, then $h_{1} (p) \sim h_{2} (p)$;
4. If $S(h_{1} (p)) = S(h_{2} (p))$ and $D(h_{1} (p)) < D(h_{2} (p))$, then $h_{1} (p) \succ h_{2} (p)$;

### Cumulative Prospect Theory

The psychological behaviors of DMs in GEDM of an epidemic are portrayed by utilizing CPT in this study. It is a descriptive model for the decision behavior of human beings under uncertainty and risk. The decision-making process is exhibited in CPT as the following two phases:

(i) Editing phase. A RP is constructed to determine the gains and losses that are represented by the difference between RP and outcome.
(ii) Evaluation phase. The edited prospects are calculated by using *value function* and *weighting function,* and the optimal alternative is selected according to the computed overall prospect values.

The value function and the weighting function are the key elements of CPT, which can explain the following five major phenomena of decision making under risk and uncertainty [27].

*Framing effects*. Equivalent formulations of a choice problem should lead to the same preference order under the hypothesis of the rational theory of choice. However, the evidence suggests that preferences change with the framing of options.

*Nonlinear preference*. It was observed that nonlinear preferences were prevalent in risky choices, for instance, the difference between probabilities of 0.99 and 1.00 has more influence on preferences than the difference between 0.10 and 0.11.

*Source dependence*. People usually prefer to bet on events within their ability rather than bet on a matched fortuitous event, although the former probability is ambiguous and the latter is clear.

*Risk seeking*. Risk seeking is universal when people must make a choice between a substantial probability of a larger loss and a sure loss. Moreover, the probability of a result with small probability and lager gains is often overestimated, which gives rise to risk-seeking choices.

*Loss aversion*. One of the basic phenomena of choice is that people are much more sensitive to losses than to equal gains.

The five phenomena mentioned above generally exist in the process of risk decision making [27]. DMs perceive gains and losses according to the corresponding RP, which in accordance with the principle of diminishing sensitivity. Hence, the value function is normally convex for losses, commonly concave for gains, and is steeper for losses than for gains in the light of the principles of framing effects and loss aversion. As a result, a two-part power function is used to express the value function as follows:

6 $$ v(z) = \left\{ {\begin{array}{*{20}c} {z^{\alpha } , \, z \ge 0} \\ { - \lambda ( - z)^{\beta } ,z < 0} \\ \end{array} } \right., $$

where $z$ is the value of deviations from the RP, $z \ge 0$ and $z < 0$ denote the gains and losses respectively. $\alpha$ and $\beta$ are the power parameters, which represent the sensitivity level of DMs with regard to gains and losses respectively, $0 \le \alpha \le 1$, $0 \le \beta \le 1$. $\lambda$ that satisfies $\lambda \ge 1$ indicates the risk-aversion degree of DMs. Generally, $\alpha = \beta = 0.88$ and $\lambda = 2.25$ [27].

A distinctive fourfold pattern of risk attitudes is presented by Kahneman and Tversky [27]: risk aversion for losses and risk seeking for gains of low probability; risk seeking for losses and risk aversion for gains. The preference of DMs related to probability of an outcome generally is nonlinear, which can be expressed by Eq. (7) that is the weighting function in CPT.

7 $$ \pi (r) = \left\{ {\begin{array}{*{20}c} {\frac{{r^{\chi } }}{{(r^{\chi } + (1 - r)^{\chi } )^{1/\chi } }},z \ge 0} \\ {\frac{{r^{\delta } }}{{(r^{\delta } + (1 - r)^{\delta } )^{1/\delta } }},z < 0} \\ \end{array} } \right., $$

where $r$ is the probability of the outcome $z$, $\chi$ and $\delta$ denote the degree of distortion on probability $r$ facing gains and losses respectively, $0 < \chi < 1$, $0 < \delta < 1$. Experimentally, $\chi = 0.61$ and $\delta = 0.69$ [27].

## The Quality Parameters for Decision Information

For the greater usage of decision information, in this section, we develop a method to measure the quality of decision information represented by PHFS that is used to estimate the proximity to its authentic value.

### Definition 6

Let a parameter that varies from 0 to 1 represent the quality of the PHFS $h(p)$. All experts provide the evaluation values of a criterion concerning an alternative in the form of PHFS, and $\overline{h}(p)$ indicates the mean PHFS of these PHFSs. The closer $h(p)$ is to the mean PHFS, the higher its quality, and the quality parameter $q$ of $h(p)$ can be defined based on Eq. (3).

8 $$ q = 1 - \frac{{d(h(p),\overline{h}(p))}}{{{\text{MAX}}\left( {\left| {h(p)} \right|,\left| {\overline{h}(p)} \right|} \right)}}, $$

where $\overline{h}(p)$ can be calculated by using the PHFWA operator and expert weights. When all the experts give the same PHFS, the quality parameter of each PHFS is best equaling 1. Moreover, we will give an example to further illustrate how the quality parameter works.

### Example 1

For a criterion related to an alternative, suppose that $h_{1} (p) = \left\{ {0.4(0.3),0.6(0.7)} \right\}$ and $h_{2} (p) = \left\{ {0.5(0.4),0.6(0.6)} \right\}$ are the evaluation values provided by expert A and expert B respectively, and the two experts have the same weights that equal to 0.5. Then, the mean PHFS is determined:

$$\overline{h}(p) = 0.5h_{1} (p) \oplus 0.5h_{2} (p) = \left\{ {0.45(0.12),0.51(0.18),0.55(0.28),0.6(0.42)} \right\}.$$

Add PHFEs to $h_{1} (p)$:

$$ h_{1} (p) = \left\{ {0.4(0),0.4(0),0.4(0.3),0.6(0.7)} \right\}. $$

Afterwards, the quality parameter $q_{1}$ can be computed:

$$ q_{1} = 1 - \frac{{d(h_{1} (p),\overline{h}(p))}}{{{\text{MAX}}\left( {\left| {h_{1} (p)} \right|,\left| {\overline{h}(p)} \right|} \right)}} = 1 - \frac{{\frac{1}{2}(0.0540 + 0.0918 + 0.0466 + 0.1680)}}{4} = 0.95495. $$

Based on quality parameters, the credibility of an expert with respect to a criterion can be represented. Considering the reliability of evaluation values makes for better decision results, and quality parameter of decision information can be used to represent or revise the expert weights.

## Probabilistic Hesitant Fuzzy Prospect Sets

This section transforms decision information in form of PHFS into the gains and losses based on CPT, and then probabilistic hesitant fuzzy prospect set (PHFPS) is established to portray the perception of DMs.

In the process of GEDM for an epidemic, different evaluation values for a criterion are usually provided. In addition, the same evaluation value may have different meanings for different DMs. More specifically, each DM has his/her expectation level for the performance of an alternative relative to a criterion, which leads to DMs’ different perceptions for the same criterion value. For instance, a medium evaluation value satisfies the DMs that have low expectation levels but can not content the DMs with high expectation levels.

The expectation levels of DMs are regarded as RPs, and the attitudes towards gains and losses are described in CPT. Moreover, Ren et al. [2] constructed hesitant fuzzy prospect decision matrix by comparing decision values and expectations of DMs. Inspired by this, we develop PHFPSs based on PHFSs and CPT, and the corresponding definition is provided as follows.

### Definition 7

Let $$h(p)$$ be a PHFS, which is used to represent the evaluation value of an alternative concerning a criterion, and $$\tilde{h}(p)$$ is the corresponding expectation level. $$\overset{\lower0.5em\hbox{$\smash{\scriptscriptstyle\smile}$}}{h} (p)$$ represents probabilistic hesitant fuzzy prospect set, and $$\overset{\lower0.5em\hbox{$\smash{\scriptscriptstyle\smile}$}}{\gamma }_{l}$$ are probabilistic hesitant fuzzy prospect elements, then the following discussions can be made.

1. When the evaluation value is better than the DM’s expectation level, which is described as $\gamma_{l} > \tilde{\gamma }_{l}$, where $\gamma_{l} \in h(p)$ and $\tilde{\gamma }_{l} \in \tilde{h}(p)$, the excessive part is considered as the gain. Then, the perceived value of $\gamma_{l}$ relative to $\tilde{\gamma }_{l}$ is defined as follows.
  9 $$ \overset{\lower0.5em\hbox{$\smash{\scriptscriptstyle\smile}$}}{\gamma }_{l} = \left( {\frac{{\gamma_{l} - \tilde{\gamma }_{l} }}{{1 - \tilde{\gamma }_{l} }}} \right)^{\alpha } . $$
2. When the evaluation value is equal to the corresponding expectation level, which can be expressed as $$\gamma_{l} = \tilde{\gamma }_{l}$$, where $$\gamma_{l} \in h(p)$$ and $$\tilde{\gamma }_{l} \in \tilde{h}(p)$$. The perceived value of $$\gamma_{l}$$ is 0, namely, $$\overset{\lower0.5em\hbox{$\smash{\scriptscriptstyle\smile}$}}{\gamma }_{l} = 0$$.
3. If the evaluation value is worse than the corresponding expectation level, this situation can be expressed by $\gamma_{l} < \tilde{\gamma }_{l}$, where $\gamma_{l} \in h(p)$ and $\tilde{\gamma }_{l} \in \tilde{h}(p)$. The perceived value of $\gamma_{l}$ can be calculated by following formulate.
  10 $$ \overset{\lower0.5em\hbox{$\smash{\scriptscriptstyle\smile}$}}{\gamma }_{l} = \frac{1}{\lambda }\left( {e^{{ - (\frac{{\tilde{\gamma }_{l} - \gamma_{l} }}{{1 - \gamma_{l} }})}} - e^{ - 1} } \right)^{\beta } , $$

where $0 \le \alpha$, $\beta \le 1$, and $\lambda \ge 1$. By the discussions mentioned above, the evaluation values are transformed into the perceived value that are all fuzzy numbers. Thus, the operations and aggregations of PHFS can be applied to PHFSPs. We separately prove the expression for the above rules by borrowing Ren et al. [2].

1. For the first rule, due to $0 \le \gamma_{l}$, $\tilde{\gamma }_{l} \le 1$, $\gamma_{l} \ge \tilde{\gamma }_{l}$ and $\tilde{\gamma }_{l} \ne 1$, the inequalities $0 \le \gamma_{l} - \tilde{\gamma }_{l} \le 1 - \tilde{\gamma }_{l} < 1$ hold. For $0 \le \alpha \le 1$ defined in the value function of CPT, $0 \le ({{\gamma_{l} - \tilde{\gamma }_{l} } \mathord{\left/ {\vphantom {{\gamma_{l} - \tilde{\gamma }_{l} } {1 - \tilde{\gamma }_{l} }}} \right. \kern-\nulldelimiterspace} {1 - \tilde{\gamma }_{l} }})^{1} \le ({{\gamma_{l} - \tilde{\gamma }_{l} } \mathord{\left/ {\vphantom {{\gamma_{l} - \tilde{\gamma }_{l} } {1 - \tilde{\gamma }_{l} }}} \right. \kern-\nulldelimiterspace} {1 - \tilde{\gamma }_{l} }})^{\alpha } \le ({{\gamma_{l} - \tilde{\gamma }_{l} } \mathord{\left/ {\vphantom {{\gamma_{l} - \tilde{\gamma }_{l} } {1 - \tilde{\gamma }_{l} }}} \right. \kern-\nulldelimiterspace} {1 - \tilde{\gamma }_{l} }})^{0} = 1$. Hence, the PHFPEs of this rule are all fuzzy numbers.
2. It is clear that 0 is one of the fuzzy numbers.
3. According to $0 \le \gamma_{l}$, $\tilde{\gamma }_{l} \le 1$, the inequalities $0 \le \tilde{\gamma }_{l} - \gamma_{l} \le 1 - \gamma_{l} < 1$ hold. For $0 \le \beta \le 1$, $0 = \left( {e^{ - 1} - e^{ - 1} } \right)^{\beta } \le \left( {e^{{ - (({{\tilde{\gamma }_{l} - \gamma_{l} } \mathord{\left/ {\vphantom {{\tilde{\gamma }_{l} - \gamma_{l} } {1 - \gamma_{l} }}} \right. \kern-\nulldelimiterspace} {1 - \gamma_{l} }}))}} - e^{ - 1} } \right)^{\beta } \le \left( {e^{0} - e^{ - 1} } \right)^{\beta } < 1$. Moreover, due to $\lambda \ge 1$, the values of $({1 \mathord{\left/ {\vphantom {1 {\lambda )}}} \right. \kern-\nulldelimiterspace} {\lambda )}}\left( {e^{{ - (({{\tilde{\gamma }_{l} - \gamma_{l} } \mathord{\left/ {\vphantom {{\tilde{\gamma }_{l} - \gamma_{l} } {1 - \gamma_{l} }}} \right. \kern-\nulldelimiterspace} {1 - \gamma_{l} }}))}} - e^{ - 1} } \right)^{\beta }$ are located in [0,1), which are fuzzy numbers.

PHFPSs consider not only vacillation and probabilistic preference of DMs but also the psychological behaviors of DMs, which makes the decision results more reasonable and practical.

### Example 2

Based on the evaluation information $$h_{1} (p) = \left\{ {0.4(0.3),0.6(0.7)} \right\}$$ in Example 1, the expert A gives his/her expectation level $$\tilde{h}_{1} (p) = \left\{ {0.5(1)} \right\}$$. Then, PHFPS $$\overset{\lower0.5em\hbox{$\smash{\scriptscriptstyle\smile}$}}{h}_{1} (p)$$ can be computed:

$$ \overset{\lower0.5em\hbox{$\smash{\scriptscriptstyle\smile}$}}{h}_{1} (p) = \left\{ {\left( {\frac{1}{2.25}\left( {{\text{e}}^{{ - (\frac{0.5 - 0.4}{{1 - 0.4}})}} - {\text{e}}^{ - 1} } \right)^{0.88} } \right)(0.3),\left( {\left( {\frac{0.6 - 0.5}{{1 - 0.5}}} \right)^{0.88} } \right)(0.7)} \right\} = \left\{ {0.23(0.3),0.24(0.7)} \right\}. $$

## The proposed GEDM Method Based on PHFPSs Under an Epidemic

In this section, a PHFPS-based MCGDM method is proposed for solving the GEDM problem of a sudden epidemic. Using the proposed GEDM method, the best epidemic emergency alternative can be selected. First, the formulation of GEDM problem under an epidemic is given. Furthermore, the revised expert weights are calculated, and then a decision-making procedure is provided to obtain the optimal emergency alternative.

### Formulation of the GEDM Problem Under an Epidemic

In the early stage of outbreak, the future evolution of the epidemic is difficult to accurately determine due to the difficulty in determining population mobility, infectivity of disease, and incubation period, etc. Hence, there are some possible states of epidemic, and the evaluation values of emergency alternatives may change with the states. The experts in infection prevention and control of an epidemic can construct the probability distribution of all the possible states to quantify the randomness based on historical experience and existing data.

For emergency alternative selection problem with PHFPSs under a major epidemic, let $M = \left\{ {1,2, \cdots m} \right\}$, $N = \left\{ {1,2, \cdots ,n} \right\}$, $O = \left\{ {1,2, \cdots ,o} \right\}$ and $C = \left\{ {1,2, \cdots ,c} \right\}$. Assume that $P = \left\{ {P_{1} ,P_{2} , \cdots ,P_{m} } \right\}$ is the finite set of alternatives, where $P_{i} (i \in M)$ denotes the $i{\text{th}}$ alternative. Let $A = \left\{ {A_{1} ,A_{2} , \cdots ,A_{n} } \right\}$ be a finite criteria set, where $A_{j} (j \in N)$ denotes the *j*th criterion; let ${\varvec{\theta}} = (\theta_{1} ,\theta_{2} , \cdots ,\theta_{n} )$ be the criterion weight vector, where $\theta_{j} (j \in N)$ is the weight of criterion $A_{j}$ such that $\sum\nolimits_{j = 1}^{n} {\theta_{j} = 1}$ and $\theta_{j} \ge 0$. The possible natural states set of the epidemic can be denoted by $B{ = }\left\{ {B_{1} ,B_{2} , \cdots ,B_{o} } \right\}$, where $B_{t} (t \in O)$ is *t*th natural state; the probability vector of natural states can be expressed by $r{ = }(r_{1} ,r_{2} , \cdots ,r_{o} )$, where $r_{t} (t \in O)$ is the probability of *t*th natural state. Assume that $L = \left\{ {L_{1} ,L_{2} , \cdots ,L_{c} } \right\}$ is the finite set of experts, where $L_{k} (k \in C)$ denotes the *k*th expert, let $\varpi = (\varpi_{1} ,\varpi_{2} , \cdots ,\varpi_{c} )$ be the expert’s weight vector, where $\varpi_{k} (k \in C)$ is the weighting of expert $L_{k}$ such that $\sum\nolimits_{k = 1}^{c} {\varpi_{k} = 1}$ and $\theta_{j} \ge 0$.

Let $\tilde{\user2{h}}_{t}^{k} {(}p{) = }\left( {\tilde{h}_{1t}^{k} (p),\tilde{h}_{2t}^{k} (p), \cdots ,\tilde{h}_{jt}^{k} (p)} \right)$ be the expectation vector of criteria, where $\tilde{h}_{jt}^{k} (p)$ is the expectation of *k*th expert corresponding to *j*th criterion under *t*th natural state. To sum up, the evaluation information is described by decision matrix ${\varvec{H}}_{{\varvec{t}}}^{{\varvec{k}}} = \left( {h_{ijt}^{k} (p)} \right)_{m \times n}$ as Eq. (12), and $h_{ijt}^{k} (p)$ denotes the evaluation value of alternative $P_{i}$ concerning criterion $A_{j}$ under natural state $B_{t}$.

11 $$ {\varvec{H}}_{t}^{{\varvec{k}}} = \left( {h_{ijt}^{k} (p)} \right)_{m \times n} = \left( {\begin{array}{*{20}c} {h_{11t}^{k} (p)} & {h_{12t}^{k} (p)} & \cdots & {h_{1nt}^{k} (p)} \\ {h_{21t}^{k} (p)} & {h_{22t}^{k} (p)} & \cdots & {h_{2nt}^{k} (p)} \\ \vdots & \vdots & \vdots & \vdots \\ {h_{m1t}^{k} (p)} & {h_{m2t}^{k} (p)} & \cdots & {h_{mnt}^{k} (p)} \\ \end{array} } \right). $$

### Revision of the Expert Weights

Expert weight represents the influence degree and the consistency of experts in the group decision-making process. In practical GEDM of an epidemic, the experts come from a variety of research fields, and there may be some difference for them in knowledge structures, practical experience, individual preference and so on. For a criterion of emergency alternatives, some experts are familiar, and while others may not be familiar. It means that the validity of each evaluation value may be different for the same criterion, it is extremely significant to identify and weaken the influence of untrusted evaluation values on decision results. Hence, determination and revision of expert weights are an important step in the GEDM of an epidemic, the accurate expert weights are conducive to the reasonable aggregation of evaluation value. The quality parameter of evaluation value is an effective tool to portray the reliability of an expert for criteria. Based on quality parameter, the expert weights can be revised to make the value of the evaluation information closer to the real situation. Revised expert weight $$\overset{\lower0.5em\hbox{$\smash{\scriptscriptstyle\frown}$}}{\varpi }_{ijt}^{k}$$ considering the quality parameter $$q_{ijt}^{k}$$ of evaluation value $$h_{ijt}^{k} (p)$$ is defined as

12 $$ \overset{\lower0.5em\hbox{$\smash{\scriptscriptstyle\frown}$}}{\varpi }_{ijt}^{k} = \frac{{\varpi_{k} q_{ijt}^{k} }}{{\sum\limits_{k = 1}^{c} {\varpi_{k} q_{ijt}^{k} } }}, $$

where $q_{ijt}^{k} = 1 - {{(d(h_{ijt}^{k} (p),\overline{h}_{ijt}^{k} (p))} \mathord{\left/ {\vphantom {{(d(h_{ijt}^{k} (p),\overline{h}_{ijt}^{k} (p))} {{\text{MAX}}(\left| {h_{ijt}^{k} (p)} \right|,\left| {\overline{h}_{ijt}^{k} (p)} \right|)}}} \right. \kern-\nulldelimiterspace} {{\text{MAX}}(\left| {h_{ijt}^{k} (p)} \right|,\left| {\overline{h}_{ijt}^{k} (p)} \right|)}})$ and $\overline{h}_{ijt}^{k} (p) = \mathop \oplus \limits_{k = 1}^{c} \varpi_{k} h_{ijt}^{k} (p)$. Significantly, the expert weights may be completely unknow, in this case, quality parameter can be used to determine the expert weights. We can assume that the expert weight $\varpi_{k}$ is equal to $1/k$. It means that the reliability of the evaluation values with respect to criteria is regarded as expert weight.

### Group Emergency Decision-Making Procedure

***Step 1.*** A decision team that consisted of *k* experts is formed, the *m* emergency alternatives, *n* evaluation criteria, and *a* natural states are determined. The weight vector of the criteria and the probabilities of natural states are obtained. Then, the criteria data in the form of PHFS is collected, and the decision matrix ${\varvec{H}}_{{\varvec{t}}}^{{\varvec{k}}} = \left( {h_{ijt}^{k} (p)} \right)_{m \times n}$ and expectation vector $\tilde{\user2{h}}_{t}^{k} {(}p{) = }\left( {\tilde{h}_{1t}^{k} (p),\tilde{h}_{2t}^{k} (p), \cdots ,\tilde{h}_{jt}^{k} (p)} \right)$ are constructed.

***Step 2.*** The expectation vector $$\tilde{\user2{h}}_{t}^{k} {(}p{) = }\left( {\tilde{h}_{1t}^{k} (p),\tilde{h}_{2t}^{k} (p), \cdots ,\tilde{h}_{jt}^{k} (p)} \right)$$ is considered as RPs, and the probabilistic hesitant fuzzy prospect decision matrix $$\overset{\lower0.5em\hbox{$\smash{\scriptscriptstyle\smile}$}}{H}_{t}^{k} = \left( {\overset{\lower0.5em\hbox{$\smash{\scriptscriptstyle\smile}$}}{h}_{ijt}^{k} (p)} \right)_{m \times n}$$ is obtained based on Definition 7, in which $$h_{ijt}^{k} (p)$$ is compared with RP.

***Step 3.*** The quality parameter $$q_{ijt}^{k}$$ is calculated by using Eq. (2) and Eq. (8), then the revised expert weight $$\overset{\lower0.5em\hbox{$\smash{\scriptscriptstyle\frown}$}}{\varpi }_{ijt}^{k}$$ is determined according to Eq. (12).

***Step 4.*** The weighted function in CPT is an effective tool to describe the uncertain risk attitudes of experts for the probabilities of possible natural states of an epidemic, the perceived probability $\pi (r_{ijt}^{k} )$ of *k*th expert to *t*th natural state is computed by using Eq. (14).

13 $$ \pi (r_{ijt}^{k} ) = \left\{ {\begin{array}{*{20}c} {\frac{{(r_{ijt}^{k} )^{\chi } }}{{((r_{ijt}^{k} )^{\chi } + (1 - (r_{ijt}^{k} ))^{\chi } )^{1/\chi } }},S(h_{ijt}^{k} (p)) > S(\tilde{h}_{jt}^{k} (p))} \\ {\frac{{(r_{ijt}^{k} )^{\delta } }}{{((r_{ijt}^{k} )^{\delta } + (1 - (r_{ijt}^{k} ))^{\delta } )^{1/\delta } }},S(h_{ijt}^{k} (p)) \le S(\tilde{h}_{jt}^{k} (p))} \\ \end{array} } \right.. $$

***Step 5.*** The overall evaluation value $Z_{ij}$ of *i*th alternative related to *j*th criterion is determined by

14 $$ Z_{ij} = Z_{ij}^{1} \oplus Z_{ij}^{2} \oplus \cdots \oplus Z_{ij}^{c} ,, $$

where $Z_{ij}^{k}$ is derived by

15 $$ Z_{ij}^{k} = \pi (r_{ij1}^{k} )\overset{\lower0.5em\hbox{$\smash{\scriptscriptstyle\frown}$}}{\varpi }_{ij1}^{k} \overset{\lower0.5em\hbox{$\smash{\scriptscriptstyle\smile}$}}{h}_{ij1}^{k} (p) \oplus \pi (r_{ij2}^{k} )\overset{\lower0.5em\hbox{$\smash{\scriptscriptstyle\frown}$}}{\varpi }_{ij2}^{k} \overset{\lower0.5em\hbox{$\smash{\scriptscriptstyle\smile}$}}{h}_{ij2}^{k} (p) \oplus \cdots \oplus \pi (r_{ijo}^{k} )\overset{\lower0.5em\hbox{$\smash{\scriptscriptstyle\frown}$}}{\varpi }_{ijo}^{k} \overset{\lower0.5em\hbox{$\smash{\scriptscriptstyle\smile}$}}{h}_{ijo}^{k} (p). $$

***Step 6.*** The final evaluation value $Y_{i}$ of *i*th alternative is obtained by

16 $$ Y_{i} = \sum\limits_{j = 1}^{n} {\theta_{j} S(Z_{ij} )} , $$

and the rankings of all alternatives are acquired according to the rule that the bigger the final evaluation value, the better the alternative is.

**Fig. 2:**
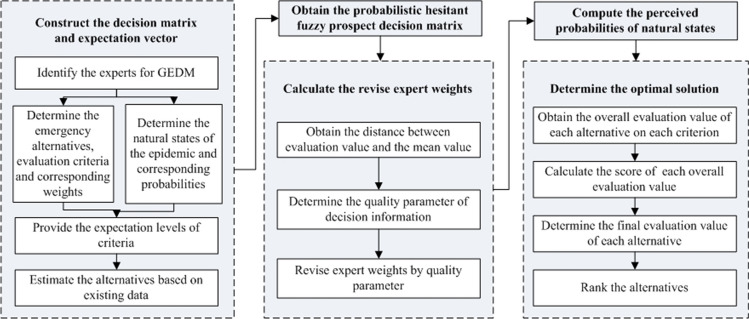
Flowchart of the proposed method

Figure 2 is presented to concisely describe the algorithm of the proposed method.

## Case Study

In this section, the EDM problem of an epidemic is taken as an example to illustrate the feasibility and practicability of the proposed method. Moreover, the comparison with other approaches and the sensitivity analysis are conducted to demonstrate the effectiveness of the proposed method.

### Implementation and Results

**Fig. 3:**
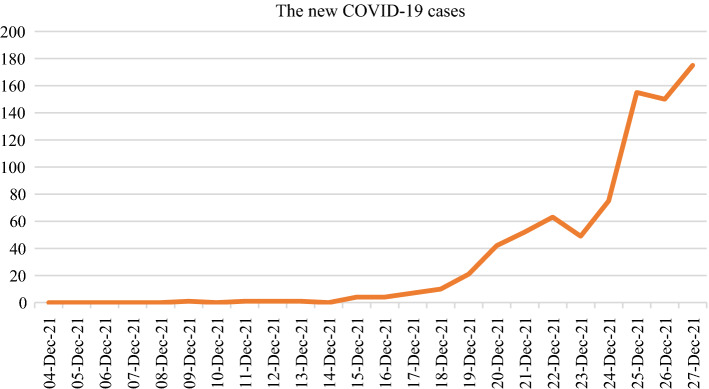
The data on the daily number of new COVID-19 cases in Xi'an

On December 9th, 2021, another wave of COVID-19 epidemic broke out in Xi'an, Shaanxi, China, and then the number of infections increased rapidly in a few days. The data on the daily number of new COVID-19 cases is shown in Fig. 3. The Delta variant, which is responsible for this outbreak, causes more infections and spreads faster than the original SARS-CoV-2 strain of the virus that cause COVID-19. Hence, the risk of COVID-19 spreading further is very high in Xi'an. There is no doubt that the economic development and human health will be greatly threatened if COVID-19 epidemic spins out of control. For slowing the spread of COVID-19 and protecting the public health from suffering a greater crisis, it is very important for the local government to make an emergency decision.

***Step 1.*** An emergency decision team composed of two experts (i.e., $L_{1}$ and $L_{2}$) and six data collectors is formed to develop and select an appreciate epidemic emergency solution, and the weighting of each expert is equal, namely, $\varpi_{1} = \varpi_{2} = 0.5$. The effect of an emergency alternative is usually different under the different states of COVID-19 in this city. According to the historical data, the decision team predicts that there may be two potential states of COVID-19 in the future shown as follows.

$B_{1}$: the confirmed cases increase slowly or no longer, the spread of COVID-19 in the city is controllable.

$B_{2}$: the confirmed cases increase rapidly, the spread of COVID-19 shows an uncontrollable trend.

By the analysis of the decision team, the probabilities of the two states are estimated, i.e., $r_{1} = 0.7$ and $r_{2} = 0.3$.

In order to ensure that the emergency solution to be implemented is more suitable, the decision team preliminarily develops four alternatives (i.e., $P_{1}$, $P_{2}$, $P_{3}$, and $P_{4}$ shown below), and determines the best one. The evaluation criteria in Table 1 are used to assess the four alternatives. The decision team provided a weight vector of evaluation criteria by the application of AHP as ${\varvec{\theta}} = (0.24,0.12,0.16,0.19,0.14,0.15)$.

$P_{1}$: the moderate lockdown policies are conducted to strike the right balance between the effects on public health and on the economy. For example, the high-risk areas are blocked, and others maintain normal production and life.

$P_{2}$: the loose lockdown policies are implemented to minimize the impacts of the epidemic on their economy.

$P_{3}$: the strictest lockdown policies are executed to cut off all unnecessary connections between the infected city and others.

$P_{4}$: the relatively strict lockdown policies (e.g., lockdown of the city, stop production, and home quarantine) are implemented to prevent the spread of the epidemic.

Based on the sufficient survey and the analysis of the decision team, the evaluation value $h_{ijt}^{k} (p)$ is determined as shown in Table 2.

**Table 2:** The evaluation values of alternatives

			Effectiveness $(A_{1} )$	Restrictiveness $(A_{2} )$	Compliance $(A_{3} )$	Emergency supplies $(A_{4} )$	Cost support $(A_{5} )$	Economic relief $(A_{6} )$
$L_{1}$	$B_{1}$	$P_{1}$	{0.3(0.3),0.5(0.7)}	{0.3(1)}	{0.7(0.6),0.8(0.4)}	{0.6(0.5),0.7(0.5)}	{0.3(0.5),0.4(0.5)}	{0.4(1)}
		$P_{2}$	{0.4(0.4),0.6(0.6)}	{0.4(0.5),0.5(0.5)}	{0.6(1)}	{0.6(1)}	{0.5(1)}	{0.4(0.5),0.5(0.5)}
		$P_{3}$	{0.4(0.2),0.5(0.8)}	{0.5(1)}	{0.5(0.4),0.6(0.6)}	{0.5(0.6),0.7(0.4)}	{0.4(0.4),0.5(0.6)}	{0.5(0.3),0.6(0.7)}
		$P_{4}$	{0.6(0.6),0.7(0.4)}	{0.3(0.3),0.4(0.7)}	{0.4(0.6),0.5(0.4)}	{0.4(0.5),0.6(0.5)}	{0.8(1)}	{0.5(0.5),0.7(0.5)}
	$B_{2}$	$P_{1}$	{0.3(0.4),0.5(0.6)}	{0.3(1)}	{0.6(0.3),0.8(0.7)}	{0.5(0.3),0.6(0.7)}	{0.3(0.5),0.4(0.5)}	{0.4(1)}
		$P_{2}$	{0.4(0.4),0.6(0.6)}	{0.4(0.5),0.5(0.5)}	{0.5(1)}	{0.4(0.6),0.6(0.4)}	{0.5(1)}	{0.4(0.5),0.5(0.5)}
		$P_{3}$	{0.5(0.6),0.7(0.4)}	{0.5(1)}	{0.5(0.5),0.6(0.5)}	{0.5(1)}	{0.4(0.4),0.5(0.6)}	{0.5(0.3),0.6(0.7)}
		$P_{4}$	{0.6(0.7),0.7(0.3)}	{0.3(0.3),0.4(0.7)}	{0.6(0.7),0.7(0.3)}	{0.3(1)}	{0.8(1)}	{0.5(0.5),0.7(0.5)}
$L_{2}$	$B_{1}$	$P_{1}$	{0.3(0.5),0.4(0.5)}	{0.3(0.7),0.5(0.3)}	{0.7(1)}	{0.8(1)}	{0.5(1)}	{0.5(1)}
		$P_{2}$	{0.4(1)}	{0.3(0.8),0.6(0.2)}	{0.5(1)}	{0.5(0.8),0.7(0.2)}	{0.5(0.3),0.6(0.7)}	{0.5(0.3),0.6(0.7)}
		$P_{3}$	{0.5(0.3),0.6(0.7)}	{0.5(1)}	{0.4(0.6),0.5(0.4)}	{0.5(0.3),0.6(0.7)}	{0.4(0.5),0.6(0.5)}	{0.5(0.2),0.7(0.8)}
		$P_{4}$	{0.5(0.4),0.7(0.6)}	{0.4(1)}	{0.5(1)}	{0.5(1)}	{0.6(0.8),0.8(0.2)}	{0.6(0.4),0.9(0.6)}
	$B_{2}$	$P_{1}$	{0.4(1)}	{0.3(0.7),0.5(0.3)}	{0.7(1)}	{0.7(1)}	{0.5(1)}	{0.5(1)}
		$P_{2}$	{0.5(1)}	{0.3(0.8),0.6(0.2)}	{0.6(1)}	{0.6(0.5),0.7(0.5)}	{0.5(0.3),0.6(0.7)}	{0.5(0.3),0.6(0.7)}
		$P_{3}$	{0.5(0.5),0.6(0.5)}	{0.5(1)}	{0.6(0.3),0.8(0.7)}	{0.5(0.6),0.6(0.4)}	{0.4(0.5),0.6(0.5)}	{0.5(0.2),0.7(0.8)}
		$P_{4}$	{0.5(0.3),0.8(0.7)}	{0.4(1)}	{0.8(1)}	{0.5(1)}	{0.6(0.8),0.8(0.2)}	{0.6(0.4),0.9(0.6)}

Each expert has the same expectation under the two states, and the expectation level $\tilde{h}_{jt}^{k} (p)$ is provided as follows.

$$ \tilde{h}_{j1}^{1} (p) = \tilde{h}_{j2}^{1} (p) = (\{ 0.7(1)\} ,\{ 0.3(1)\} ,\{ 0.5(1)\} ,\{ 0.8(1)\} ,\{ 0.6(1)\} ,\{ 0.5(1)\} ), $$

$$ \tilde{h}_{j1}^{1} (p) = \tilde{h}_{j2}^{1} (p) = (\{ 0.8(1)\} ,\{ 0.3(1)\} ,\{ 0.43(1)\} ,\{ 0.6(1)\} ,\{ 0.6(1)\} ,\{ 0.7(1)\} ). $$

***Step 2. ***The probabilistic hesitant fuzzy prospect value $$\overset{\lower0.5em\hbox{$\smash{\scriptscriptstyle\smile}$}}{h}_{ijt}^{k} (p)$$ is obtained as shown in Table 3.

**Table 3:** The probabilistic hesitant fuzzy prospect value $$\overset{\lower0.5em\hbox{$\smash{\scriptscriptstyle\smile}$}}{h}_{ijt}^{k} (p)$$

			Effectiveness $(A_{1} )$	Restrictiveness $(A_{2} )$	Compliance $(A_{3} )$	Emergency supplies $(A_{4} )$	Cost support $(A_{5} )$	Economic relief $(A_{6} )$
$L_{1}$	$B_{1}$	$P_{1}$	{0.11(0.3),0.16(0.7)}	{0.00(1)}	{0.45(0.6),0.64(0.4)}	{0.13(0.5),0.18(0.5)}	{0.15(0.5),0.18(0.5)}	{0.23(1)}
		$P_{2}$	{0.13(0.4),0.20(0.6)}	{0.18(0.5),0.33(0.5)}	{0.24(1)}	{0.13(1)}	{0.22(1)}	{0.23(0.5),0.00(0.5)}
		$P_{3}$	{0.13(0.2),0.16(0.8)}	{0.33(1)}	{0.00(0.4),0.24(0.6)}	{0.10(0.6),0.18(0.4)}	{0.18(0.4),0.22(0.6)}	{0.00(0.3),0.24(0.7)}
		$P_{4}$	{0.20(0.6),0.00(0.4)}	{0.00(0.3),0.18(0.7)}	{0.23(0.6),0.00(0.4)}	{0.08(0.5),0.13(0.5)}	{0.54(1)}	{0.00(0.5),0.45(0.5)}
	$B_{2}$	$P_{1}$	{0.11(0.4),0.16(0.6)}	{0.00(1)}	{0.24(0.3),0.64(0.7)}	{0.10(0.3),0.13(0.7)}	{0.15(0.5),0.18(0.5)}	{0.23(1)}
		$P_{2}$	{0.13(0.4),0.20(0.6)}	{0.18(0.5),0.33(0.5)}	{0.00(1)}	{0.08(0.6),0.13(0.4)}	{0.22(1)}	{0.23(0.5),0.00(0.5)}
		$P_{3}$	{0.16(0.6),0.00(0.4)}	{0.33(1)}	{0.00(0.5),0.24(0.5)}	{0.10(1)}	{0.18(0.4),0.22(0.6)}	{0.00(0.3),0.24(0.7)}
		$P_{4}$	{0.20(0.7),0.00(0.3)}	{0.00(0.3),0.18(0.7)}	{0.24(0.7),0.45(0.3)}	{0.07(1)}	{0.54(1)}	{0.00(0.5),0.45(0.5)}
$L_{2}$	$B_{1}$	$P_{1}$	{0.07(0.5),0.08(0.5)}	{0.00(0.7),0.33(0.3)}	{0.52(1)}	{0.54(1)}	{0.22(1)}	{0.16(1)}
		$P_{2}$	{0.08(1)}	{0.00(0.8),0.47(0.2)}	{0.16(1)}	{0.22(0.8),0.30(0.2)}	{0.22(0.3),0.00(0.7)}	{0.16(0.3),0.20(0.7)}
		$P_{3}$	{0.10(0.3),0.13(0.7)}	{0.33(1)}	{0.28(0.6),0.16(0.4)}	{0.22(0.3),0.00(0.7)}	{0.18(0.5),0.00(0.5)}	{0.16(0.2),0.00(0.8)}
		$P_{4}$	{0.10(0.4),0.18(0.6)}	{0.18(1)}	{0.16(1)}	{0.22(1)}	{0.00(0.8),0.54(0.2)}	{0.20(0.4),0.70(0.6)}
	$B_{2}$	$P_{1}$	{0.08(1)}	{0.00(0.7),0.33(0.3)}	{0.52(1)}	{0.30(1)}	{0.22(1)}	{0.16(1)}
		$P_{2}$	{0.10(1)}	{0.00(0.8),0.47(0.2)}	{0.34(1)}	{0.00(0.5),0.30(0.5)}	{0.22(0.3),0.00(0.7)}	{0.16(0.3),0.20(0.7)}
		$P_{3}$	{0.10(0.5),0.13(0.5)}	{0.33(1)}	{0.34(0.3),0.68(0.7)}	{0.22(0.6),0.00(0.4)}	{0.18(0.5),0.00(0.5)}	{0.16(0.2),0.00(0.8)}
		$P_{4}$	{0.10(0.3),0.00(0.7)}	{0.18(1)}	{0.68(1)}	{0.22(1)}	{0.00(0.8),0.54(0.2)}	{0.20(0.4),0.70(0.6)}

***Step 3. ***The quality parameter $q_{ijt}^{k}$ is computed by using Eq. (2) and Eq. (8) as exhibited in Table 4.

**Table 4:** The quality parameter $q_{ijt}^{k}$ of the evaluation value $h_{ijt}^{k} (p)$

			Effectiveness $(A_{1} )$	Restrictiveness $(A_{2} )$	Compliance $(A_{3} )$	Emergency supplies $(A_{4} )$	Cost support $(A_{5} )$	Economic relief $(A_{6} )$
$L_{1}$	$B_{1}$	$P_{1}$	0.954	0.948	0.988	0.984	0.985	0.987
		$P_{2}$	0.989	0.947	0.988	0.885	0.963	0.944
		$P_{3}$	0.968	1.000	0.945	0.918	0.955	0.959
		$P_{4}$	0.907	0.997	0.969	0.991	0.848	0.925
	$B_{2}$	$P_{1}$	0.977	0.948	0.991	0.987	0.985	0.987
		$P_{2}$	0.991	0.947	0.987	0.927	0.963	0.944
		$P_{3}$	0.918	1.000	0.930	0.925	0.944	0.959
		$P_{4}$	0.889	0.997	0.980	0.973	0.848	0.925
$L_{2}$	$B_{1}$	$P_{1}$	0.978	0.996	0.896	0.900	0.937	0.988
		$P_{2}$	0.960	0.954	0.987	0.989	0.993	0.930
		$P_{3}$	0.968	1.000	0.957	0.919	0.951	0.948
		$P_{4}$	0.909	0.972	0.930	0.940	0.979	0.905
	$B_{2}$	$P_{1}$	0.949	0.996	0.949	0.943	0.937	0.988
		$P_{2}$	0.952	0.954	0.988	0.909	0.993	0.930
		$P_{3}$	0.921	1.000	0.907	0.997	0.939	0.948
		$P_{4}$	0.878	0.972	0.864	0.977	0.979	0.905

Then, the revised expert weight $$\overset{\lower0.5em\hbox{$\smash{\scriptscriptstyle\frown}$}}{\varpi }_{ijt}^{k}$$ is calculated according to Eq. (12) as shown in Table 5.

**Table 5:** The revised expert weight $$\overset{\lower0.5em\hbox{$\smash{\scriptscriptstyle\frown}$}}{\varpi }_{ijt}^{k}$$

			Effectiveness $(A_{1} )$	Restrictiveness $(A_{2} )$	Compliance $(A_{3} )$	Emergency supplies $(A_{4} )$	Cost support $(A_{5} )$	Economic relief $(A_{6} )$
$L_{1}$	$B_{1}$	$P_{1}$	0.494	0.488	0.522	0.522	0.512	0.500
		$P_{2}$	0.507	0.498	0.500	0.472	0.492	0.504
		$P_{3}$	0.500	0.500	0.497	0.500	0.501	0.503
		$P_{4}$	0.500	0.507	0.511	0.513	0.464	0.506
	$B_{2}$	$P_{1}$	0.507	0.488	0.511	0.511	0.512	0.500
		$P_{2}$	0.510	0.498	0.500	0.505	0.492	0.504
		$P_{3}$	0.499	0.500	0.506	0.481	0.501	0.503
		$P_{4}$	0.503	0.507	0.531	0.499	0.464	0.506
$L_{2}$	$B_{1}$	$P_{1}$	0.506	0.512	0.478	0.478	0.488	0.500
		$P_{2}$	0.493	0.502	0.500	0.528	0.508	0.496
		$P_{3}$	0.500	0.500	0.503	0.500	0.499	0.497
		$P_{4}$	0.500	0.493	0.489	0.487	0.536	0.494
	$B_{2}$	$P_{1}$	0.493	0.512	0.489	0.489	0.488	0.500
		$P_{2}$	0.490	0.502	0.500	0.495	0.508	0.496
		$P_{3}$	0.501	0.500	0.494	0.519	0.499	0.497
		$P_{4}$	0.497	0.493	0.469	0.501	0.536	0.494

***Step 4. ***The perceived probability $\pi (r_{ijt}^{k} )$ is determined by utilizing Eq. (13) and listed in Table 6.

**Table 6:** The perceived probability $\pi (r_{ijt}^{k} )$

			Effectiveness $(A_{1} )$	Restrictiveness $(A_{2} )$	Compliance $(A_{3} )$	Emergency supplies $(A_{4} )$	Cost support $(A_{5} )$	Economic relief $(A_{6} )$
$L_{1}$	$B_{1}$	$P_{1}$	0.782	0.782	0.804	0.782	0.782	0.782
		$P_{2}$	0.782	0.804	0.804	0.782	0.782	0.782
		$P_{3}$	0.782	0.804	0.804	0.782	0.782	0.804
		$P_{4}$	0.782	0.804	0.782	0.782	0.804	0.804
	$B_{2}$	$P_{1}$	0.436	0.436	0.480	0.436	0.436	0.436
		$P_{2}$	0.436	0.480	0.436	0.436	0.436	0.436
		$P_{3}$	0.436	0.480	0.480	0.436	0.436	0.480
		$P_{4}$	0.436	0.480	0.480	0.436	0.480	0.480
$L_{2}$	$B_{1}$	$P_{1}$	0.782	0.804	0.804	0.804	0.782	0.782
		$P_{2}$	0.782	0.804	0.804	0.782	0.782	0.782
		$P_{3}$	0.782	0.804	0.804	0.782	0.782	0.782
		$P_{4}$	0.782	0.804	0.804	0.782	0.804	0.804
	$B_{2}$	$P_{1}$	0.436	0.480	0.480	0.480	0.436	0.436
		$P_{2}$	0.436	0.480	0.480	0.480	0.436	0.436
		$P_{3}$	0.436	0.480	0.480	0.436	0.436	0.436
		$P_{4}$	0.436	0.480	0.480	0.436	0.480	0.480

***Step 5. ***The overall evaluation value $Z_{ij}$ is given based on Eq. (14) and Eq. (15), i.e.,

$$ Z_{i = 1,j = 1} = \{ 0.109(0.06),0.113(0.06),0.120(0.09),0.124(0.09),0.203(0.14),0.208(0.14),0.213(0.21),0.217(0.21)\} , $$

$$ Z_{i = 1,j = 2} = \{ 0.000(0.49),0.094(0.21),0.153(0.21),0.233(0.009)\} , $$

$$ Z_{i = 1,j = 3} = \{ 0.536(0.18),0.612(0.12),0.613(0.42),0.676(0.28)\} , $$

$$ Z_{i = 1,j = 4} = \{ 0.370(0.15),0.374(0.35),0.385(0.15),0.389(0.35)\} , $$

$$ Z_{i = 1,j = 5} = \{ 0.219(0.25),0.225(0.25),0.230(0.25),0.235(0.25)\} , $$

$$ Z_{i = 1,j = 6} = \{ 0.232(1)\} , $$

$$ Z_{i = 2,j = 1} = \{ 0.129(0.16),0.147(0.24),0.161(0.24),0.178(0.36)\} , $$

$$ Z_{i = 2,j = 2} = \left\{ {\begin{array}{*{20}c} {0.120(0.16),0.162(0.16),0.189(0.16),0.228(0.16),0.246(0.04),0.282(0.04),0.305(0.04),0.321(0.04),} \\ {0.338(0.04),0.353(0.04),0.374(0.04),0.404(0.04),0.418(0.01),0.446(0.01),0.464(0.01),0.490(0.01)} \\ \end{array} } \right\}, $$

$$ Z_{i = 2,j = 3} = \{ 0.246(1)\} , $$

$$ Z_{i = 2,j = 4} = \{ 0.157(0.24),0.166(0.16),0.192(0.06),0.200(0.04),0.225(0.24),0.233(0.16),0.256(0.06),0.264(0.04)\} , $$

$$ Z_{i = 2,j = 5} = \{ 0.262(0.09),0.220(0.21),0.185(0.21),0.139(0.49)\} , $$

$$ Z_{i = 2,j = 6} = \left\{ {\begin{array}{*{20}c} {0.097(0.0225),0.108(0.0525),0.117(0.0525),0.128(0.1225),0.148(0.0225),0.159(0.0525),0.167(0.0525),0.177(0.1225),} \\ {0.186(0.0225),0.196(0.0525),0.204(0.0525),0.214(0.1225),0.232(0.0225),0.242(0.0525),0.249(0.0525),0.259(0.1225)} \\ \end{array} } \right\}, $$

$$ Z_{i = 3,j = 1} = \left\{ {\begin{array}{*{20}c} {0.109(0.012),0.115(0.012),0.120(0.028),0.121(0.048),0.126(0.028),0.127(0.048),0.132(0.112),0.138(0.112),} \\ {0.142(0.018),0.147(0.018),0.152(0.042),0.153(0.072),0.157(0.042),0.159(0.072),0.163(0.168),0.169(0.168)} \\ \end{array} } \right\}, $$

$$ Z_{i = 3,j = 2} = \{ 0.404(1)\} , $$

$$ Z_{i = 3,j = 3} = \left\{ {\begin{array}{*{20}c} {0.156(0.024),0.206(0.036),0.211(0.024),0.245(0.036),0.258(0.036),0.290(0.11),0.294(0.036),} \\ {0.332(0.084),0.336(0.11),0.364(0.084),0.376(0.084),0.402(0.126),0.406(0.084),0.441(0.126)} \\ \end{array} } \right\}, $$

$$ Z_{i = 3,j = 4} = \{ 0.061(0.168),0.093(0.112),0.112(0.252),0.142(0.168),0.148(0.072),0.177(0.048),0.194(0.108),0.222(0.072)\} , $$

$$ Z_{i = 3,j = 5} = \left\{ {\begin{array}{*{20}c} {0.111(0.04),0.122(0.06),0.131(0.06),0.141(0.09),0.148(0.04),0.158(0.06),0.166(0.06),0.176(0.13),} \\ {0.186(0.06),0.194(0.06),0.203(0.09),0.210(0.04),0.219(0.06),0.227(0.06),0.236(0.09)} \\ \end{array} } \right\}, $$

$$ Z_{i = 3,j = 6} = \left\{ {\begin{array}{*{20}c} {0.000(0.0576),0.036(0.0144),0.063(0.0144),0.065(0.1344),0.097(0.0036),0.098(0.0336),0.106(0.1344),0.124(0.0336),} \\ {0.138(0.0336),0.156(0.0084),0.163(0.0336),0.164(0.3136),0.193(0.0084),0.194(0.0784),0.217(0.0784),0.245(0.0196)} \\ \end{array} } \right\}, $$

$$ Z_{i = 4,j = 1} = \left\{ {\begin{array}{*{20}c} {0.040(0.0336),0.061(0.0144),0.073(0.0504),0.086(0.0784),0.094(0.0216),0.107(0.0336),0.118(0.1176),0.121(0.0504),} \\ {0.138(0.0504),0.141(0.0216),0.152(0.0756),0.164(0.1176),0.170(0.0324),0.183(0.0504),0.193(0.1764),0.211(0.0756)} \\ \end{array} } \right\}, $$

$$ Z_{i = 4,j = 2} = \{ 0.118(0.09),0.160(0.21),,0.187(0.21),0.226(0.49)\} , $$

$$ Z_{i = 4,j = 3} = \{ 0.395(0.42),0.442(0.18),0.328(0.28),0.380(0.12)\} , $$

$$ Z_{i = 4,j = 4} = \{ 0.180(0.5),0.197(0.5)\} , $$

$$ Z_{i = 4,j = 5} = \{ 0.373(0.64),0.489(0.16),0.553(0.16),0.635(0.04)\} , $$

$$ Z_{i = 4,j = 6} = \left\{ {\begin{array}{*{20}c} {0.134(0.04),0.250(0.04),0.313(0.06),0.319(0.04),0.405(0.06),0.410(0.04),0.413(0.06),0.460(0.06),} \\ {0.491(0.06),0.532(0.06),0.534(0.09),0.538(0.06),0.597(0.09),0.600(0.06),0.634(0.09),0.683(0.09)} \\ \end{array} } \right\}. $$

***Step 6. ***The final evaluation value $Y_{i}$ is computed by using Eq. (4) and (16) as $Y_{1} { = }0.2902$, $Y_{2} { = }0.1964$, $Y_{3} { = }0.2086$, and $Y_{4} { = }0.2887$. Then, the ranking of alternatives is determined, i.e., $P_{1} \succ P_{4} \succ P_{3} \succ P_{2}$, where “$\succ$” denotes “be better than”. It can be seen that $P_{1}$ is the optimal emergency alternative. Thus, $P_{1}$ is going to be implemented for halting the spread of COVID-19.

### Comparison with Other Approaches

To further demonstrate the effectiveness and feasibility of the proposed method, the following several comparisons are conducted.

1. The proposed method is compared with the aggregation operator-based multiattribute group decision making (MAGDM) method [54], PHFS-based TOPSIS method [56], and PHFS-based VIKOR method [57], which are developed based on the strict assumption that DMs are completely rational.
2. The proposed method is compared with the extended PHFS-based TODIM method [58], which considers the DMs’ psychologies of loss aversion and diminishing sensitivity.
3. The research suggests that the DM’s expectation is universal and has an irresistible effect on the decision results [27]. In order to further illustrate that it is necessary to consider the DMs’ expectations in GEDM. We extend the aggregation operator-based MAGDM method [54] by considering the PRs, and the proposed method is compared with the extended aggregation operator-based method.
4. The proposed method is compared with the proposed method that excludes the quality parameters for decision information by assuming that all quality parameters in the proposed method are 1.

In order to ensure the comparability and rationality of decision results, the same original decision information in the abovementioned case study is used to conduct the comparison analysis. The final evaluation values and rankings by all approaches are shown in Table 7. It can be seen from Table 7 that the rankings of alternatives are quite diverse according to different methods, so it is difficult to compare and analyze the decision results. To this end, we utilize rank-biased overlap model proposed in [59] to depict the difference and similarity between the decision results. Rank-biased overlap model can also satisfy our expectation that the top-ranking items are more important compared to lower ranking items, the implementation process is as follows.

**Table 7:** The final evaluation values and rankings by several methods

Cases	Used methods	The final evaluation values				Rank
		$P_{1}$	$P_{2}$	$P_{3}$	$P_{4}$	
Case 1: Consider DMs’ psychologies	The proposed method	0.2902	0.1964	0.2086	0.2887	$P_{1} \succ P_{4} \succ P_{3} \succ P_{2}$
Case 2: Without considering DMs’ psychologies	The aggregation operator-based MAGDM method	0.5153	0.5152	0.5477	0.5949	$P_{4} \succ P_{3} \succ P_{1} \succ P_{2}$
Case 3: Without considering DMs’ psychologies	The PHFS-based TOPSIS method	0.2892	0.4394	0.5205	0.6961	$P_{4} \succ P_{3} \succ P_{2} \succ P_{1}$
Case 4: Without considering DMs’ psychologies	The PHFS-based VIKOR method	0.9057	0.5595	0.2289	0.2110	$P_{4} \succ P_{3} \succ P_{2} \succ P_{1}$
Case 5: Consider DMs’ psychologies except expectations	The PHFS-based TODIM method	0.0000	0.7001	0.3195	1.0000	$P_{4} \succ P_{2} \succ P_{3} \succ P_{1}$
Case 6: Without considering DMs’ psychologies except expectations	The extended aggregation operator-based MAGDM method	0.3136	0.2550	0.2429	0.2948	$P_{1} \succ P_{4} \succ P_{2} \succ P_{3}$
Case 7: Consider DMs’ psychologies and without considering quality of decision information	The proposed method without considering quality of decision information	0.2917	0.1960	0.2087	0.2927	$P_{4} \succ P_{1} \succ P_{3} \succ P_{2}$

***Step 1. ***Let *T* and *U* be two rankings shown in Table 7, and let $T_{y}$ be the element at rank $y$ in list $T$. $T_{f:u}$ denotes the set of the elements from position $f$ to position $u$ in list $T$, $f$ and $u$ are regarded as the depths of the set $T_{:f}$ and set $T_{:u}$ respectively. The intersection of list $T$ and list $U$ to depth $\zeta$ is expressed by $V_{T,U,\zeta } = T_{:\zeta } \cap U_{:\zeta }$, and $W_{T,U,\zeta }$ is considered as the size of this intersection such that $W_{T,U,\zeta } = \left| {V_{T,U,\zeta } } \right|$.

***Step 2.*** $R_{T,U,\zeta }$ represents the agreement of list $T$ and list $U$ at depth $\zeta$, and can be calculated based on the proportion of $T$ and $U$ that are overlapped, $R_{T,U,\zeta } = {{W_{T,U,\zeta } } \mathord{\left/ {\vphantom {{W_{T,U,\zeta } } \zeta }} \right. \kern-\nulldelimiterspace} \zeta }$.

***Step 3.*** $E$ denotes the evaluation depth, and the average overlap of $T$ and $U$ can be defined as:

17 $$ AO(T,U,E){ = }\frac{1}{E}\sum\limits_{\zeta = 1}^{E} {R_{T,U,\zeta } } $$

***Step 4. ***The degree of similarity between different rankings is computed based on Eq. (17) and the results are as shown in Table 8.

**Table 8:** The degree of rank similarity between different methods

	Case 1	Case 2	Case 3	Case 4	Case 5	Case 6	Case 7
Case 1	1.000	0.625	0.542	0.542	0.542	0.917	0.750
Case 2	0.625	1.000	0.917	0.917	0.792	0.542	0.875
Case 3	0.542	0.917	1.000	1.000	0.875	0.542	0.792
Case 4	0.542	0.917	1.000	1.000	0.875	0.542	0.792
Case 5	0.542	0.792	0.875	0.875	1.000	0.542	0.792
Case 6	0.917	0.542	0.542	0.542	0.542	1.000	0.667
Case 7	0.750	0.875	0.792	0.792	0.792	0.667	1.000

#### Example 3

Suppose that $T$ is $P_{1} \succ P_{4} \succ P_{3} \succ P_{2}$, which is the ranking of alternatives under Case 1 in Table 7. Let $U$ be the ranking under Case 2 such that $P_{4} \succ P_{3} \succ P_{1} \succ P_{2}$. Then, the agreement of $T$ and $U$ can be calculated: $R_{T,U,1} = {{W_{T,U,1} } \mathord{\left/ {\vphantom {{W_{T,U,1} } 1}} \right. \kern-\nulldelimiterspace} 1} = {{\left| {V_{T,U,\zeta } } \right|} \mathord{\left/ {\vphantom {{\left| {V_{T,U,\zeta } } \right|} 1}} \right. \kern-\nulldelimiterspace} 1} = {{\left| {T_{:1} \cap U_{:1} } \right|} \mathord{\left/ {\vphantom {{\left| {T_{:1} \cap U_{:1} } \right|} 1}} \right. \kern-\nulldelimiterspace} 1} = {{\left| {\{ P_{1} \} \cap \{ P_{4} \} } \right|} \mathord{\left/ {\vphantom {{\left| {\{ P_{1} \} \cap \{ P_{4} \} } \right|} 1}} \right. \kern-\nulldelimiterspace} 1} = {0 \mathord{\left/ {\vphantom {0 1}} \right. \kern-\nulldelimiterspace} 1} = 0$, $R_{T,U,2} = {{\left| {\{ P_{1} ,P_{4} \} \cap \{ P_{4} ,P_{3} \} } \right|} \mathord{\left/ {\vphantom {{\left| {\{ P_{1} ,P_{4} \} \cap \{ P_{4} ,P_{3} \} } \right|} 2}} \right. \kern-\nulldelimiterspace} 2} = {1 \mathord{\left/ {\vphantom {1 2}} \right. \kern-\nulldelimiterspace} 2} = 0.5$ similarly, $R_{T,U,3} = 1$, $R_{T,U,4} = 1$. After that, we get the degree of similarity between both rankings as: $AO(T,U,4) = \frac{1}{4}(0 + 0.5 + 1 + 1) = 0.625$.

From the degree of similarity listed in Table 8, we can conduct the further analysis. Then, several conclusions and the main advantages of the proposed method can be summarized as follows.

1. From Table 8, the similarity measures of the proposed method with the aggregation operator-based MAGDM method, the PHFS-based TOPSIS method and the PHFS-based VIKOR method are 0.625, 0.542 and 0.542 respectively. The main reason for the difference among rankings obtained by these approaches is that their assumptions are different, our method assumes that DMs are bounded rational. More specifically, DMs prefer the alternative that is close to their expectations, and the performance of a criterion will be distorted when the evaluation values are inconsistent with the corresponding expectation level. When there is a big gap between the evaluation value of an alternative and the corresponding expectation level, the psychology of loss aversion is considered, which may lead to the low priority of the alternative. Moreover, the degree of rank similarity between the results of the latter three methods is very high, which illustrates the compared methods are effective. In the process of emergency decision making, considering DMs’ psychological factors makes the decision results better reflect humans’ way of thinking in real–world situations. However, these three methods that are compared with the proposed method are presented based on the strict assumption that the DMs are completely rational, which results in the distinct ranking results.
2. The TODIM method determines the alternatives’ rankings according to the dominance degree of each alternative over the others. However, the proposed method estimates the performances of criteria according to the corresponding expectation levels. The TODIM method considers the DMs’ psychological behaviors except expectations, the degree of rank similarity between the proposed method and the extended PHFS-based TODIM method is 0.542, which shows the importance of considering expectations. The degree of rank similarity between the TODIM method and the aggregation operator-based MAGDM method, the PHFS-based TOPSIS method and the PHFS-based VIKOR method are 0.792, 0.875, and 0.875, respectively. It indicates that the psychologies of loss aversion and diminishing sensitivity have an effect on decision results in GEDM.
3. The degree of rank similarity between the proposed method and the extended aggregation operator-based MAGDM method is 0.917. The ranking results determined by the proposed method are remarkably similar to the approach that takes the DMs’ expectations into account. This further proves that DMs’ expectations have a considerable influence on decision results, and it is necessary to consider expectation levels of DMs in the process of GEDM.
4. The degree of rank similarity between the proposed method and the proposed method that excludes the quality parameters is 0.750. This shows that the quality of decision information has a significant effect on the decision results, DMs should pay attention to the data structure of evaluation information. In the actual emergency decision-making process, it is difficult to evaluate the credibility of the evaluation values provided by the experts. Our method takes full advantage of decision information and weakens the effect of outliers on decision results, which makes the decision results more reasonable.

### Sensitivity Analysis of the Loss Aversion Parameter

**Fig. 4:**
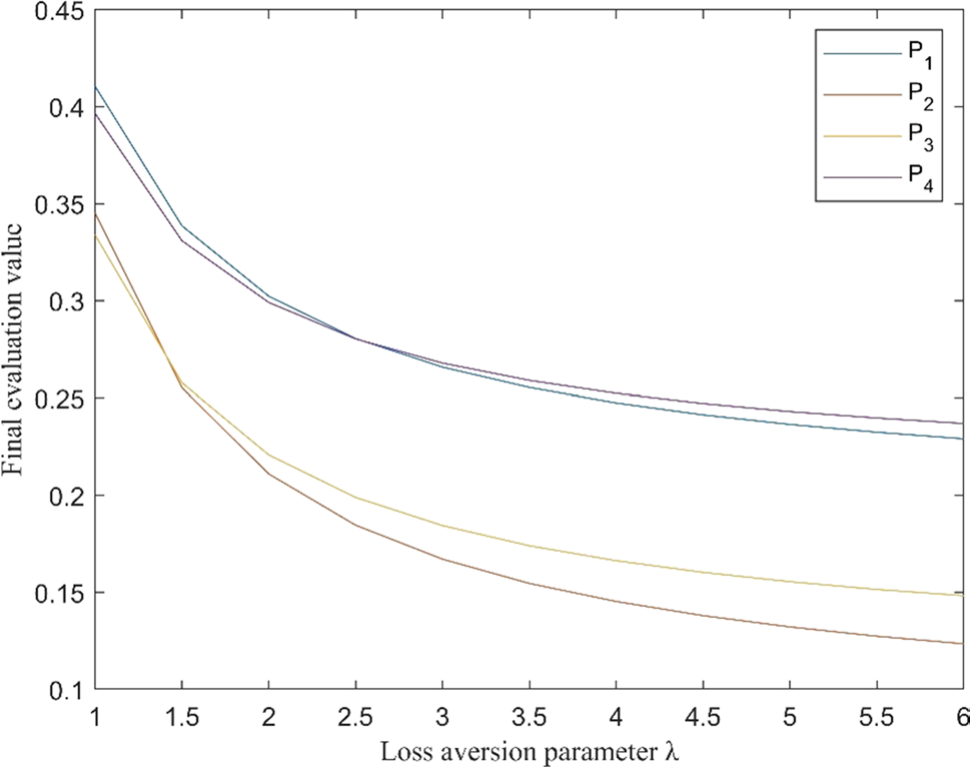
Final evaluation value $Y_{i}$ with different values of loss aversion parameter $\lambda$

In the practical process of GEDM, the DMs’ psychological behaviors, especially loss aversion, have great impact on the decision results. Thus, it is significant to conduct a sensitivity analysis of the loss aversion parameter $\lambda$. The final evaluation values of emergency alternatives concerning different levels of loss aversion are graphically portrayed in Fig. 4.

It is observed from Fig. 4 that final evaluation values decrease with the increase of loss aversion parameter. However, emergency alternatives differ from one another for the degree of dropping. $P_{1}$ is the best alternative when $\lambda = 1$, and the ranking of $P_{1}$ is declining along with the rising $\lambda$. In more detail, for the losses relative to the expectation levels, the performances of $P_{1}$ and $P_{2}$ are worse than $P_{4}$ and $P_{3}$ respectively. Thus, the underestimation of experts with respect to losses of $P_{1}$ and $P_{2}$ increases with $\lambda$ quickly, which results in the decreasing ranking. The rankings of all alternatives remain unchanged when $\lambda$ is greater than 2.53, and $P_{4}$ is the optimal solution.

### Managerial Implications

Under the epidemic scenarios, an effective and practicable GEDM method can remarkably reduce the loss of property and life. However, using an unreliable GEDM method without taking the information quality and the psychological factors into consideration may result in the deterioration of epidemic prevention. This study emphasizes the importance of considering the quality of decision information and the psychologies of DMs, and several theoretical and practical implications are as follows.

1. The proposed PHFPS-based MCGDM method is the first to consider the quality of decision information represented by PHFSs, which has a significant influence on the decision results. Our method makes full use of evaluation information given by the decision team. More specifically, the quality parameter of evaluation information helps DMs discover the true value of decision information rather than the existing GEDM methodologies and thus makes a significant contribution to a appreciate outcome.
2. The proposed method fully considers and addresses the psychological behaviors of experts under risk and uncertainty in the GEDM process of an epidemic, compared with the current GEDM studies that do not take DMs’ psychologies into consideration. This is a significant difference between our method and the current GEDM frameworks, our method is closer to the real-world situation. In particular, the decision results are determined based on RPs, which is good for a more expectant and desirable decision. Moreover, the comparison in Sect. 7.2 and the sensitivity analysis conducted in Sect. 7.3 highlight again the effect of psychologies of DMs on the final outcomes. Hence, this study can serve as a useful reference to other researchers to explore the GEDM under the hypothesis that DMs are bounded rationality.
3. Based on the actual situation and existing literature, we summarize the evaluation criteria and corresponding definitions for selecting the optimal emergency solution of an epidemic. DMs can add or delete evaluation criteria according to their own requirements.

This study not only adds to academic knowledge by providing a novel GEDM method of an epidemic, but also offer reference for DMs when emergencies occur.

## Conclusions

The existing GEDM approaches mainly focus on how to cope with the incomplete and inadequate decision information in emergencies or select the ideal emergency alternatives. They neglect the importance of DMs’ psychological behaviors in the GEDM process, and few studies pay attention to the psychologies of DMs. Moreover, the uncertainty of emergency events’ future evolutions, the vagueness of decision information and the DMs’ hesitations should be considered. Most important of all, epidemics bring great harm to human health and economic development, however, few studies develop the complete and appropriate EDM frameworks of epidemics. Based on such problems, this study proposes a GEDM method of epidemics based on PHISs and CPT considering information quality.

Compared with the existing methods, the proposed method presents PHFPSs to portray the DMs’ psychological behaviors and the vagueness of decision information, which can better retain decision information. There are significant differences between the developed method and the other versions that use CPT. It takes the future evolutions of emergency events into consideration, which is closer to practical GEDM of epidemics and easy for DMs to accept and apply. In addition, this study also takes the quality of decision information into account, which is conducive to more reliable use of evaluation information.

The proposed GEDM method have been applied to a real COVID-19 epidemic example, comparison with other approaches demonstrates feasibility and validity. Limitations are also presented in the proposed method. The criteria values and weights are complete in this method. However, in some GEDM problems, the criteria values and weights may be partially known. Thus, we shall make our method obtain better applicability by considering the incomplete criteria values and weights. Additionally, in the context of group decision making, consensus reaching is conducive to yielding a collective solution with a high degree of acceptability [60]. Therefore, consensus building for GEDM is considered as the direction that required further study.

## Data Availability

The datasets used or analyzed during the current study are available from the corresponding author on reasonable request.

## Abbreviations

EDM: Emergency decision making
DMs: Decision makers
GEDM: Group emergency decision making
CPT: Cumulative prospect theory
PHFS: Probabilistic hesitant fuzzy set
PHFPS: Probabilistic hesitant fuzzy prospect set
COVID-19: The coronavirus disease 2019
EER: Epidemic emergency response
MCGDM: Multicriteria group decision making
TOPSIS: Similarity to ideal solution
TODIM: The interactive and multiple attribute decision making
PHFE: Probabilistic hesitant fuzzy element
RP: Reference point
PHFWA: Probabilistic hesitant fuzzy weighted averaging
